# The Cortical Acto-Myosin Network: From Diffusion Barrier to Functional Gateway in the Transport of Neurosecretory Vesicles to the Plasma Membrane

**DOI:** 10.3389/fendo.2013.00153

**Published:** 2013-10-21

**Authors:** Andreas Papadopulos, Vanesa M. Tomatis, Ravikiran Kasula, Frederic A. Meunier

**Affiliations:** ^1^Queensland Brain Institute, The University of Queensland, St Lucia Campus, Brisbane, QLD, Australia

**Keywords:** cortical actin, myosin, regulated exocytosis, cdc42, phosphoinositides, secretory vesicles

## Abstract

Dysregulation of regulated exocytosis is linked to an array of pathological conditions, including neurodegenerative disorders, asthma, and diabetes. Understanding the molecular mechanisms underpinning neuroexocytosis including the processes that allow neurosecretory vesicles to access and fuse with the plasma membrane and to recycle post-fusion, is therefore critical to the design of future therapeutic drugs that will efficiently tackle these diseases. Despite considerable efforts to determine the principles of vesicular fusion, the mechanisms controlling the approach of vesicles to the plasma membrane in order to undergo tethering, docking, priming, and fusion remain poorly understood. All these steps involve the cortical actin network, a dense mesh of actin filaments localized beneath the plasma membrane. Recent work overturned the long-held belief that the cortical actin network only plays a passive constraining role in neuroexocytosis functioning as a physical barrier that partly breaks down upon entry of Ca^2+^ to allow secretory vesicles to reach the plasma membrane. A multitude of new roles for the cortical actin network in regulated exocytosis have now emerged and point to highly dynamic novel functions of key myosin molecular motors. Myosins are not only believed to help bring about dynamic changes in the actin cytoskeleton, tethering and guiding vesicles to their fusion sites, but they also regulate the size and duration of the fusion pore, thereby directly contributing to the release of neurotransmitters and hormones. Here we discuss the functions of the cortical actin network, myosins, and their effectors in controlling the processes that lead to tethering, directed transport, docking, and fusion of exocytotic vesicles in regulated exocytosis.

## Introduction

**Figure 1:**
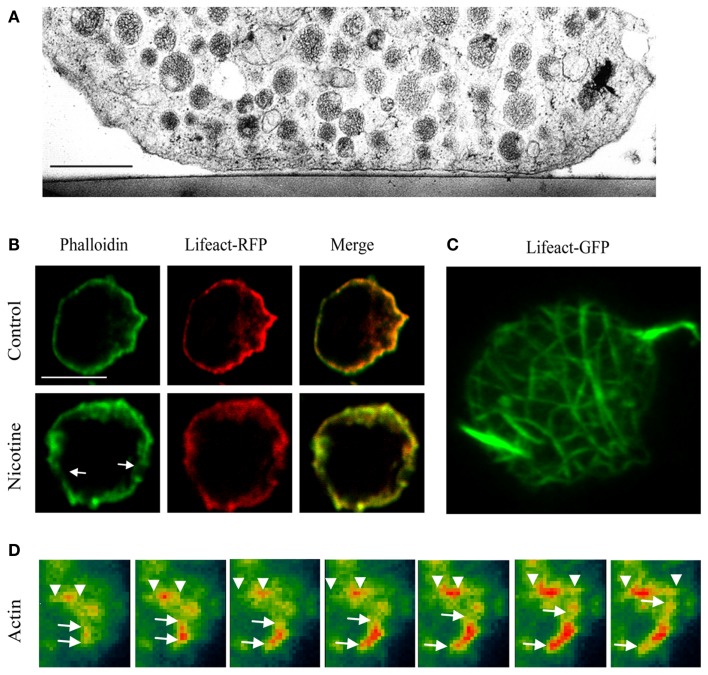
**Imaging the actin network in neurosecretory cells**. **(A)** Electron micrograph of a bovine chromaffin cell region attached to the thermanox support. Note the presence of a filamentous cortical region that is devoid of SG. Bar, 1 μm [adapted from Ref. (19)]. **(B)** Confocal images showing the mid section of bovine chromaffin cells expressing lifeact-RFP and counter stained with FITC-conjugated phalloidin in the presence or absence of nicotine (50 μM). **(C)** Maximum intensity projection of the footprint of a chromaffin cell. **(D)** TIRF images showing actin lengthening in a chromaffin cell expressing lifeact-GFP (pseudocolor) after the addition of PI3Kδ inhibitor IC87114. **(B–D)** Adapted from Ref. (2).

Regulated exocytosis relies on the timely fusion of secretory vesicles or granules (SVs/SGs) with the plasma membrane. For this to occur, SVs need to be mobilized, translocated, docked, and primed at the plasma membrane. Translocation, docking/priming, and fusion of SGs rely on dynamic changes in the cortical actin network, a dense mesh of filamentous actin underneath the plasma membrane (Figures 1A–C) that is controlled by actin effectors and myosin motor proteins. The thick actin ring of the cortical actin network can be visualized in chromaffin cells by staining actin with a variety of methods ranging from classical immunofluorescence to phalloidin (covalently linked to fluorophores), a fungal alkaloid that preferentially binds actin filaments (Figures 1B,C). More recently, the development of lifeact-GFP, a 17-residue peptide from *S. cerevisiae* that selectively binds to actin without affecting neuroexocytosis (1, 2), has allowed the probing of the dynamic changes occurring during stimulation of exocytosis on the cortical actin network by time-lapse imaging (Figures 1C,D). Following secretagogue stimulation the cortical actin ring fragments, coinciding with a decrease in cortical F-actin labeling (Figure 1B). This process is Ca^2+^-dependent and involves actin-severing proteins such as scinderin (3–6). Although actin reorganization helps vesicles reach the plasma membrane (7), F-actin also serves as an anchoring point for SGs and provides tracks for their directed motion toward fusion sites (8). Molecular motors associated with F-actin, such as myosins (9), are involved in additional functions (2, 10).

In nerve terminals, actin is a well-known modulator of neurotransmitter release. Actin is involved in synaptic vesicle mobilization as well as axonal vesicle trafficking and synaptic plasticity (11). It is the most abundant cytoskeletal protein in synapses and is highly enriched in dendritic spines, whose formation is initiated by dendritic filopodia formation (12–15), an actin-driven process facilitated by the action of myosin X (16, 17). Neurotransmitter release at central synapses is regulated by actin and depolymerization of F-actin by latrunculin A was found to transiently enhance neurotransmitter release indicating a restraining role of F-actin in active zones (18).

## New Roles for Actin in Exocytosis

The cortical actin network plays an important and well-described role during vesicle exocytosis (5, 7, 9, 10), and in recent years new functions for actin and its associated proteins have emerged (2, 9, 10, 20–24). Ca^2+^-dependent reorganization and remodeling of the cortical actin network help vesicles move toward the plasma membrane by partial disassembly of the cortical layer (Figure 1B) (3, 6). At the same time, this remodeling provides tracks that extend further toward the center of the cell allowing the mobilization of SGs from the reserve pool (25) to their docking and fusion sites at the plasma membrane (4, 26, 27). Ca^2+^ regulates the cortical F-actin disassembly in chromaffin cells via two pathways (28, 29). The first involves stimulation-induced influx of extracellular Ca^2+^ through Ca^2+^ channels and results in activation of scinderin and ensuing F-actin severing. The second pathway is triggered by Ca^2+^ release from intracellular stores (30) and can be induced in the absence of secretagogue stimulation, by phorbol esters (3). Here actin disassembly is achieved through protein kinase C (PKC) activation followed by myristoylated alanine-rich C kinase substrate (MARCKS) phosphorylation that inhibits its F-actin-binding and cross-linking properties (28). The cortical actin network provides a layered structure that retains 2–4% of the total vesicles in close proximity to the cell surface that contribute to the burst of catecholamine release at the onset of stimulation (26, 31, 32). Indeed the majority of SGs in the vicinity of the plasma membrane are tethered to the cortical actin network (6), and newly arriving vesicles are also caught in this dense mesh of F-actin (33). Other studies point to the existence of F-actin cages that organize the SNARE proteins SNAP25 and syntaxin-1 as well as L- and P/Q-type calcium channels, creating sites in the cortical actin network where SGs fuse preferentially (34). Consistent with these data, studies using total internal reflection fluorescence (TIRF) microscopy revealed that vesicle motion becomes restricted in the vicinity of the plasma membrane (35, 36). Interestingly, both actin depolymerization (37) and N-WASP- and Cdc42-dependent actin polymerization (Figure 1D) potentiate exocytosis (2, 38). While these results may appear contradictory, such opposing role for actin is not unlikely. Partial actin depolymerization helps SGs to cross the actin layer that acts as barrier, and the remaining (10) as well as newly forming actin fibers provide tracks for vesicles to reach the plasma membrane (2, 38). The balance between actin polymerization and depolymerization is likely regulated by scinderin acting as a molecular switch capable of inducing both actin polymerization and depolymerization (39). An important link connecting membranes and actin during exocytosis is the glycerophospholipid phosphatidylinositol 4,5-bisphosphate (PIP_2_). Although it is only a minor component of cellular membranes, microdomains, and clusters of PIP_2_ play a crucial role in exocytosis. PIP_2_ is known to control actin polymerization by modulating the activity and targeting of actin regulatory proteins (40). PIP_2_ involvement in SNARE-mediated exocytosis, i.e., its Ca^2+^-dependent interaction with synaptotagmin-1 and syntaxin, has been described in numerous studies (41–44). Decreased levels of PIP_2_ in the brain and impairment of its synthesis in nerve terminals lead to early postnatal lethality and synaptic defects in mice, including decreased frequency of miniature currents, enhanced synaptic depression, and a smaller ready release pool of synaptic vesicles, delayed endocytosis, and slower recycling kinetics (45). The formation of PIP_2_ microdomains at syntaxin-1A clusters with docked SGs seems to be required for Ca^2+^-dependent exocytosis (46). Both PIP_2_ and syntaxin-1A have been found in punctate nanoclusters in isolated PC12 cell plasma membrane sheets, and similar PIP_2_ clusters in PC12 cells have been reported to link synaptotagmin-1 and syntaxin-1A, thus providing a platform for SV recruitment (46, 47). Likewise, the clustering of syntaxin-1A in model membranes has been shown to be modulated by PIP_2_ (48). PIP_2_ also plays a role in regulated exocytosis by controlling several proteins involved in modifying the actin cytoskeleton (40), as well as stimulating actin polymerization (49). PIP_2_ binds scinderin in a Ca^2+^ and pH-dependent manner (50). PIP_2_ binding inhibits scinderin-induced actin depolymerization (51, 52), as well as the ADF/cofilin actin-severing activity (53) thereby promoting actin polymerization. A transient increase in PIP_2_ levels is sufficient to promote the mobilization and recruitment of SVs to the plasma membrane via Cdc42-mediated actin reorganization (2). PIP_2_ therefore links exocytosis and the actin cytoskeleton by coordinating the actin-based delivery of SVs to the plasma membrane (2). Likewise, decreasing PIP_2_ levels in neuroendocrine cells by either ATP depletion or sequestering PIP_2_ rapidly reduces the amount of cortical F-actin (54). In a similar study, nanomolar interaction of HIV-1 transcriptional activator with PIP_2_ was found to prevent the actin reorganization necessary for bringing SVs to the plasma membrane and severely impaired neurosecretion in PC12 and chromaffin cells (55). Another actin-binding protein that PIP_2_ has been found to interact with is vilin, with PIP_2_-vilin association inhibiting actin depolymerization and enhancing actin cross-linking (56). The interplay of Rho GTPases such as Cdc42, RhoA, and Rac with PIP_2_ and other actin regulatory proteins controls Ca^2+^-regulated exocytosis in chromaffin cells (9, 22). Other small GTPases implicated in regulated secretion in neurosecretory and endocrine cells are Arf6 (57), Rab27A (58) as well as RalA and Rab3A. RalA has not only been shown to tether insulin granules to R- and L-type calcium channels (59) but also binds to the exocyst complex and regulates filopodia formation linking morphological changes and regulated exocytosis (60). RalA, which is present in GLUT4 vesicles in adipocytes, also interacts with the exocyst complex and its activation is required for insulin-stimulated GLUT4 trafficking. Impairment in the function of RalA in these cells attenuated insulin-stimulated glucose transport. RalA also interacts with Myo1C acting as a cargo receptor for this motor protein (61). In addition RalA has been found to control SG exocytosis in PC12 cells by interacting with phospholipase D1. It is activated during exocytosis and the expression of a constitutively active mutant was found to enhance neuroexocytosis whereas expression of an inactive mutant or silencing resulted in reduced secretion (62). Of the four homologs (A/B/C/D) Rab3A is the best characterized (63). Rab3A is involved in the late steps of exocytosis. Early studies showed that Rab3A is associated with SG in bovine chromaffin cells and rat PC12 cells (64, 65). Overexpression of Rab3A mutant proteins defective in either GTP hydrolysis or in guanine nucleotide-binding inhibited exocytosis (66). Similarly the perfusion of Rab3A and various guanine nucleotides into chromaffin cells resulted in delayed catecholamine secretion suggesting a negative regulatory role in secretion (67). Rab3A plays a role in vesicle priming, where it is involved in Munc13-1 activation and interacts with Munc18-1 to regulate priming and fusion (68). Furthermore, Munc13 and Rab3A localize in the acrosomal region in human sperm, where they stimulate acrosomal exocytosis and play an important role in membrane docking (69). In human spermatozoa Rab3A and Rab27 act in a cascade that regulates dense core granule exocytosis (70). Rab3 interaction with Munc18 has also been shown to regulate SG density at the periphery of PC12 cells (71) and Rab3 guanine cycling is required for Munc18-dependent SG docking (72). However, the high level of redundancy between the four Rab3 isoforms makes it difficult to fully assess their individual contributions and the lack of an obvious exocytic phenotype in double and triple knock-out animals points to a regulatory but not essential role of Rab3A in exocytosis (73).

A number of new functions are now being attributed to the interplay between actin and various myosins. Non-muscle myosin II, and the unconventional myosins 1c/e, Va, and VI are involved in different stages during regulated exocytosis of SGs.

## Myosins

**Figure 2:**
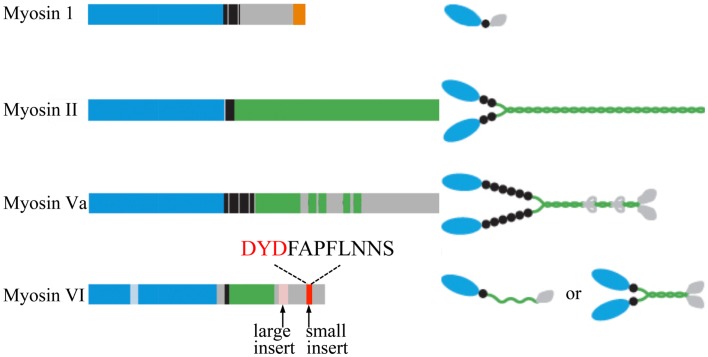
**Schematic diagrams of the myosin heavy chains involved in regulated exocytosis**. All myosins consist of a head (motor) domain (blue), a neck that contains one or more IQ motifs for light chain and CaM binding (black), and a tail domain with coiled-coil regions (green) and membrane/cargo-binding domains (orange). The small insert of myosin VI, shown to be essential for the tethering of SGs to the cortical actin network, and the DYD-Src phosphorylation motif are highlighted. Cargo-binding induced dimerization of myosin VI is likely to be mediated by the coiled-coil regions and the cargo-binding domains. Adapted from Ref. (83).

Myosins are a 17-member superfamily of actin-based molecular motor proteins (74) that are involved in many aspects of eukaryotic cell functions, including cell movement, establishment of cell shape and polarity (75–80), and vesicular trafficking (61, 81). Myosin function is not limited to that of a molecular motor, as myosins also regulate actin polymerization, serve as molecular anchors (33), and even play a role in signal transduction (82, 83). All myosins contain a heavy chain with a conserved ∼80 kDa N-terminal catalytic domain that includes the ATPase activity and actin-binding regions (Figure 2) (84). This domain is followed by an α-helical neck region containing one or more IQ motifs that allow binding of light chains and calmodulin (CaM). The C-terminal myosin tail contains cargo/membrane-binding domains, kinase activity, and/or mediates heavy chain dimerization depending on the myosin class (Figure 2) (83, 85).

### Myosin I

**Figure 3:**
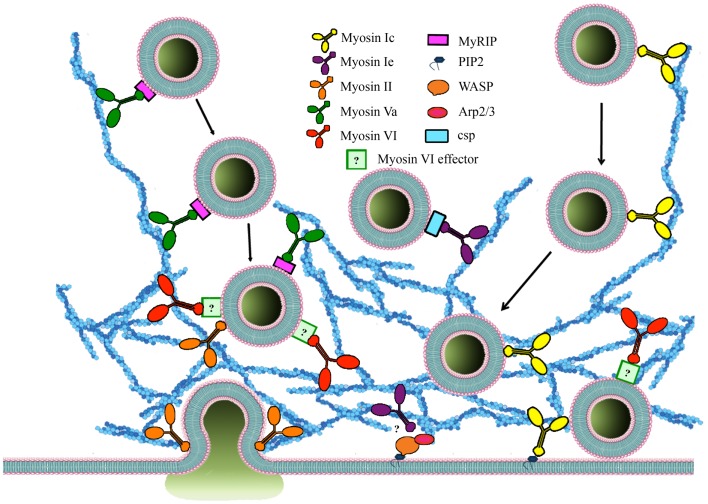
**The roles of myosins and accessory proteins involved in regulated exocytosis**. Myosins are involved in several steps of regulated exocytosis. Myosin 1C (yellow), myosin 1E (burgundy), myosin II (orange), and myosin Va (green) are involved in secretory vesicle transport. In contrast, myosin VI (red) recruits SGs to the cortical actin network. Myosin 1C interacts with SG through cysteine string proteins and myosin Va binds to MyRIP (purple) on the membrane of SGs. Myosin 1C can be recruited to membranes through PIP_2_ interaction. The effector that mediates binding between myosin VI and SGs (light green) is currently unknown. Myosin 1E is also involved in regulating actin polymerization through interaction with WASP/Arp 2/3. Cdc42 as well WASP/Arp 2/3 regulate actin polymerization in an activity-dependent manner. Myosin II also regulates size and duration of fusion pore opening.

Myosin I (Figure 2) is a single-headed membrane-associated protein that is expressed in all eukaryotic cells (84). Although there is currently no evidence for myosin I involvement in neurosecretion two isoforms of the human myosin 1C gene (86), myosin 1C (Myo1C), and myosin 1E (Myo1E) have been implicated in regulated exocytosis. All members of this unconventional myosin family interact with actin through their catalytic head domain (87). Myo1C is also capable of binding phosphoinositides (88) (Figure 3), thereby linking the actin cytoskeleton to the plasma membrane (89). Myo1C is recruited to GLUT4-containing vesicles that undergo regulated exocytosis in 3T3-L1 adipocytes in an insulin-dependent manner, and is involved in their transport to the plasma membrane (Figure 3) (61, 81). In addition, Myo1C also tethers GLUT4-containing vesicles to the cortical actin network (Figure 3) underneath the plasma membrane in response to insulin (90), and promotes GLUT4 insertion to the plasma membrane by fusion (91), thereby regulating glucose uptake in adipose and muscle tissue (92). Myo1C is required for vascular endothelial growth factor receptor-2 (VEGFR2) delivery to the cell surface and for angiogenic signaling (93). VEGF stimulation promotes the recruitment of VEGFR2 to Myo1C and its delivery to the cell surface (93).

In *Xenopus* oocytes Myo1E, the only long-tailed myosin 1 class motor protein has been found to rapidly relocate from the cytosol to cortical SGs upon secretagogue stimulation and to bind to cysteine string proteins, components of cortical SGs that mediate vital steps in regulated exocytosis (94) by interacting with SNAP25 and the calcium sensor synaptotagmin 9 in pancreatic β-cells (95). While cortical granule exocytosis is enhanced by overexpression of Myo1E it is inhibited by injection of Myo1E antibodies (94). Myo1E has also been implicated in the recruitment of several actin-binding proteins leading to N-WASP recruitment and Arp2/3-mediated actin polymerization (Figure 3) (96).

### Myosin II

Class II myosins are most abundant in muscle cells where their main function is to generate mechanical force. Non-muscle cells also contain a subset of myosin II molecules with distinct functionality. They all consist of two heavy chains (230 kDa), two regulatory light chains, and two essential light chains (Figure 2). In addition to actin cross-linking, bundling, and contractile properties, myosin II is known to regulate actin polymerization and is therefore linked to a great number of functions in eukaryotic cells including motility, adhesion (97), and regulated exocytosis (24, 98, 99). Non-muscle myosin II has been implicated in vesicle transport through the actin cytoskeleton (Figure 3). Expression of an inactive non-phosphorylatable regulatory light chain mutant myosin II fused to GFP drastically impairs granule mobility and influences actin dynamics, similar to blebbistatin treatment (100).

There is mounting evidence that myosin II is involved in controlling fusion pore dynamics and release kinetics (Figure 3). Expression of non-phosphorylatable regulatory light chain mutant myosin II that produces an inactive protein alters single vesicle fusion kinetics and slows fusion pore expansion (23, 24). Similarly, the release kinetics of fluorescently tagged tissue plasminogen activator and brain-derived neurotrophic (BDNF) factor are prolonged following overexpression of a wild-type form of the myosin II regulatory light chain and shortened by overexpression of a dominant-negative form (101). The use of a green fluorescent pH-sensitive protein (pHluorin) targeted inside the SVs revealed that the altered kinetics of release were caused by changes in the duration of fusion pore opening. Additional evidence indicates that myosin II affects catecholamine release by directly controlling the size of the fusion pore and the duration of its opening (20). Actin cortex disassembly elicited by high frequency stimulation promotes full fusion of SGs – an effect blocked by pharmacological inhibition of myosin II or myosin light chain (MLC) by preventing the fusion pore dilation (102). Inhibition of either actin polymerization with cytochalasin D or myosin II function with blebbistatin also slowed fusion pore expansion and increased its lifetime, suggesting that the interplay between actin and myosin II can accelerate catecholamine release (20). Similar results indicating that myosin II activity maintains an open fusion pore were obtained in exocrine pancreatic cells where myosin II (blebbistatin) and MLC (ML-9) inhibition did not alter the number of fusion events but resulted in a decreased fusion pore lifetime (103).

It has been suggested that myosin II contractility could also help to squeeze secretory cargo out of vesicles surrounded by an actin coat once they are connected to the plasma membrane through a fusion pore (104). Fusion pore opening and closing might not be enough to release large cargo from SVs and myosin II might provide an active extrusion mechanism (104). The direct involvement of MLC and myosin II was also observed in GLUT4-containing vesicle fusion following insulin-stimulated glucose uptake in 3T3-L1 adipocytes. Only active phosphorylated myosin II was recruited to GLUT4 vesicles in an activity-dependent manner. Interestingly, insulin specifically stimulates the myosin IIA isoform via MLC kinase phosphorylation of MLC (105, 106). Myosin II inhibition also increases the distance of SGs from the plasma membrane, and promotes the retraction of the cytoskeleton, suggesting its involvement in the final approach of vesicles toward the plasma membrane (107).

Myosin II involvement in integrin-mediated cell adhesion and exocytosis has been linked to changes in cell adhesion properties (108, 109). Glucose stimulation of pancreatic β-cells promotes the remodeling of integrin focal adhesions and phosphorylation of focal adhesion kinases and myosin II (108, 109). As myosin II is one of the main substrates of Rho-kinase 1/2, which stimulates myosin–actin interactions and induces reorganization of the actin cytoskeleton, this activity could modulate SG translocation and cargo release in response to secretagogue stimulation.

### Myosin Va

Myosin Va (Figure 2) has been implicated in exocytosis and vesicle movement to the cell periphery. In melanocytes, in a complex with Rab27a and melanophilin, myosin Va regulates melanosome transport to the plasma membrane (110, 111). In pancreatic β-cells myosin Va also functions in the transport to and retention of insulin granules at the cortical actin network under stimulated conditions as well as their secretion (112– 114). In neurosecretory cells, myosin Va is associated with SGs and plays distinctive roles during SG exocytosis (25, 115). Firstly, it assists the membrane remodeling required for SG maturation by promoting the removal of the transmembrane protein furin from maturing SGs (116). Secondly, in a complex with the SG-associated small GTPase Rab27 and its effector MyRIP, myosin Va regulates the interaction of SGs with the cortical actin network (Figure 3) (58). This complex has been implicated in exocytosis of SGs by modulating the transport of SGs and their retention in the cortical actin network on their way to the plasma membrane (117, 118). The interaction between myosin Va and MyRIP facilitates the dissociation of SGs from microtubules, enhancing their directed motion and the probability of SG docking to the plasma membrane (118). As a conventional processive molecular motor, myosin Va moves selective cargo along actin filaments (117). This feature strongly supports the key role of this protein in the translocation and tethering of SGs to the cell periphery. Blocking myosin Va function reduces the immobilization periods of SGs thereby decreasing the density of docked SGs near the plasma membrane and their exocytosis (117, 119). In resting conditions, myosin Va forms a stable complex with synaptic vesicle membrane proteins, synaptobrevin II, and synaptophysin (120). This complex is rapidly disassembled upon Ca^2+^ increase in either intact nerve endings or *in vitro* assays (120). In chromaffin cells, influx of Ca^2+^ dissociates myosin V from chromaffin vesicles supporting a role for Ca^2+^ in the regulation of transient interactions between myosin V and its cargo (25). Furthermore, when an antibody against myosin V head was introduced in permeabilized chromaffin cells after a first stimulation of 40 s, the secretory response to a second stimulation several minutes after the first one, was greatly reduced. This points to a role for myosin V in providing SVs for the refilling of the release-ready pool following stimulation (25). The role of Ca^2+^ as a regulator of the interaction between myosin V and its cargo has also been demonstrated in melanosomes and *Xenopus* egg extracts (121). Released CaM activates CaM kinase II (CaMK-II), a myosin Va binding partner (122). CaMK-II activation leads to myosin Va phosphorylation and the release of melanosomes from F-actin (121). Similarly, microinjection of CaM antibodies into chromaffin cells resulted in reduced catecholamine output in response to stimulation (123). Ca^2+^-regulated phosphorylation of myosin Va is believed to represent a universal mechanism that regulates the association between myosin Va and its cargo. These observations suggest that by regulating the interaction between myosin V and SGs, Ca^2+^ could also control the association between SGs and actin during SG mobilization in the cortical region (124). Importantly, the Ca^2+^-regulated attachment/release of myosin Va from SGs could be finely coordinated by other molecular motors, such us myosin VI (33). This cooperative model would allow a highly organized and controlled mechanism that regulates SG transport, retention, and anchoring and ultimately SG fusion with the plasma membrane.

### Myosin VI

Another member of the myosin family, myosin VI is critical for SV recruitment to the cortical actin network (Figure 2). The cellular functions of myosin VI are attributed to its unique ability to generate movement from the plus to the minus end of actin filaments. Myosin VI has an additional unique 53 aa insert, the “reverse gear,” between the motor domain and the neck region that has been predicted to be responsible for this exceptional inverted movement directionality (Figure 2) (125, 126). Interestingly, this insert binds CaM even though it does not contain a recognizable IQ-CaM motif (127). The tail domain region is the most variable amongst the myosin VI isoforms. Four alternatively spliced isoforms are generated due to the presence of a large insert (21–31 aa), a small insert (9 aa), no insert, or both inserts in this domain (Figure 2) (128, 129).

The function of myosin VI depends on the ability of its cargo-binding domain (CBD) region to interact with different binding partners that target myosin VI to specific cellular compartments (130). Myosin VI undergoes cargo-mediated dimerization a potential regulatory pathway for all myosins (131, 132). Myosin VI has been linked to clathrin- and non-clathrin-mediated endocytosis, as well as maintenance of Golgi organization and cell polarity. The large and no insert isoforms are the main isoforms mediating these functions (128, 133–137). Myosin VI has also been implicated in autophagy (138), stereocilia maintenance (139), spermatid individualization (140–142), nuclear transcription (143), and cell–cell contacts (144, 145). Evidence of a role of myosin VI in secretion were highlighted by Warner et al. (146) using immortalized cells from Snell’s waltzer mice, a strain of myosin VI knock-out mice (146–148). Immortalized fibroblastic cells from these mice have a reduced Golgi complex (∼40% smaller in comparison with that in normal cells) that is accompanied by a similar reduction in constitutive secretion (146). The down-regulation of myosin VI expression using small interfering RNA selectively reduces the secretion of prostate-specific antigen and vascular endothelial growth factor in the prostate cancer cell line LNCaP (149). Myosin VI together with its binding partner optineurin, regulates the final stage of constitutive exocytosis by mechanically controlling the formation of the fusion pore between the SV and the plasma membrane in HeLa cells (150). Less is known about the role of myosin VI in the nervous system (151). Myosin VI is widely and highly expressed in the brain; it is found in synapses and enriched at the postsynaptic density (151). In hippocampal neurons, myosin VI forms a complex with α-amino-3-hydroxy-5-methyl-4-isoxazolepropionic acid receptor (AMPAR), AP-2 and synaptic-associated protein 97 (SAP-97), and mediates AMPAR clathrin-mediated endocytosis. Importantly, myosin VI function underpins hippocampal neurons synapses and dendritic spines formation (151). Other work supports the role of myosin VI in neurotransmission by demonstrating that myosin VI; together with its binding partner GIPC1 is necessary for BDNF-TrkB-mediated synaptic plasticity (152).

Myosin VI has a very slow rate of release of ADP from its nucleotide-binding pocket, which therefore slows the dissociation of myosin VI from actin (153–155). Studies carried out in Snell’s waltzer mice have shown that myosin VI allows the formation, maturation, and function of sensory hair cells by mediating the attachment of membrane compartments to the F-actin cytoskeleton (148). Together these lines of evidence point toward the possibility that myosin VI could regulate neuroexocytosis by anchoring/recruiting SVs to the actin network before they undergo fusion with the plasma membrane. Although little is known about the precise molecular mechanism(s) underpinning this role, the function of myosin VI in regulated in exocytosis in PC12 cells has been questioned (156). However, *Drosophila* mutants lacking myosin VI display altered neuromuscular junction morphology and synaptic vesicle localization resulting in impaired synaptic plasticity (157). Myosin VI could therefore mediate the mobilization of synaptic vesicles from different functional pools, by a yet to be elucidated mechanism. We recently described a novel role for the myosin VI small insert isoform (Figure 2) in regulated exocytosis in PC12 cells (33). Using purified SGs in a pull-down approach followed by mass spectrometry, we identified myosin VI as a cytosolic protein that interacts with SGs in a Ca^2+^-dependent manner. We found that myosin VI maintains an active pool of SGs near the plasma membrane by tethering them to the cortical actin network (Figure 3). This allows the replenishment of the pool of SGs near the plasma membrane and is key to sustaining exocytosis during long periods of stimulation (33). Interestingly, we found that c-Src phosphorylation in a DYD motif located in the CBD of myosin VI small insert is one of the mechanisms controlling its function in regulated neuroexocytosis (33). The mechanisms that target myosin VI to SGs and the regulation of the isoform specific tethering function still need to be elucidated.

There are several other members of the myosin family that could potentially be involved in regulated secretion, including myosin X, a motor protein found predominantly at the tip of filopodia of many cell types including neurons (16, 17). Filopodia are important precursors for dendritic spine and synapse formation but more work is needed to assess whether neurosecretion can occur in these structures.

## Conclusion

Understanding the detailed roles of myosins and other accessory proteins in regulated exocytosis is challenging. Although a great deal is known about the involvement of these proteins and their effectors during the different stages of secretion, there is still no comprehensive model of the interplay of the different myosin isoforms, e.g., the transition from myosin Va-mediated directed transport to myosin VI-dependent recruitment to the cortical actin network. Common pathways that are shared by other cellular functions, such as adhesion or migration should also be explored further. Future work should therefore aim at combining *in vitro* techniques with live cell microscopy experiments in order to explore the complex interplay between the different myosin molecular motors during neuroexocytosis. In particular, it will be necessary to address the nature of the pathways, which coordinate and control myosin functions in order to achieve such precise spatio-temporal trafficking of SVs en route to fusion with the plasma membrane.

## Conflict of Interest Statement

The authors declare that the research was conducted in the absence of any commercial or financial relationships that could be construed as a potential conflict of interest.
